# Effects of RuPeng15 Powder (RPP15) on Monosodium Urate Crystal-Induced Gouty Arthritis in Rats

**DOI:** 10.1155/2015/527019

**Published:** 2015-06-28

**Authors:** Y.-Y. Kou, Y.-F. Li, M. Xu, W.-Y. Li, M. Yang, R.-L. Li

**Affiliations:** ^1^Pharmacology Department, Medical College, Qinghai University, China; ^2^Pharmacy Department, Qinghai Red Cross Hospital, Xining 810000, China; ^3^Gastroenterology Department, Qinghai Red Cross Hospital, Xining 810000, China

## Abstract

RuPeng15 Powder (RPP15) is a herbal multicompound remedy that originates from traditional Tibetan medicine and possesses antigout, anti-inflammatory, and antihyperuricemic properties based on the traditional conceptions. The present study was undertaken to evaluate the therapeutic effect of PRP15 in rat gouty arthritis induced by monosodium urate (MSU) crystals. In the present study, we found that treatment with RPP15 (0.4, 0.8, and 1.2 g/kg) in rats with gouty arthritis induced by MSU crystals significantly attenuated the knee swelling. Histomorphometric and immunohistochemistry analyses revealed that MSU-induced inflammatory cell infiltration and the elevated expressions of nuclear transcription factor-*κ*B p65 (NF-*κ*B p65) in synovial tissues were significantly inhibited, and enzyme-linked immunosorbent assay (ELISA) result showed that MSU-induced high levels of tumor necrosis factor-alpha (TNF-*α*), interleukin-1 beta (IL-1*β*), and interleukin-8 (IL-8) in synovial fluid were reduced by treatment with RPP15 (0.4, 0.8, and 1.2 g/kg). We conclude that RPP15 may be a promising candidate for the development of a new treatment for gout and its activity of antigout may be partially related to inhibiting TNF-*α*, IL-1*β*, IL-8, and NF-*κ*B p65 expression in the synovial tissues.

## 1. Introduction

Acute gouty arthritis is characterized by the deposition of monosodium urate monohydrate (MSU) crystals in the inflamed joints clinically in the acute form, and it is associated with intense pain and enhanced vascular permeability evidenced by edema and erythema that may extend beyond the joint margin [1, 2]. Many events can set off acute gouty attacks, including overindulgence in alcohol, metabolic stresses such as those that accompany acute myocardial infarctions or surgery, or, most predictably, major shifts in serum uric acid levels leading to resorption of MSU crystals, such as that occurring after starting urate-lowering therapy [3, 4]. The stereotypical features of gouty arthritis is that urate crystals are directly activated with all of the major synovial cell types and result in the infiltration of neutrophils in both the inflamed joint fluid and the synovial membrane [1, 5, 6]. Activated neutrophils release proteolytic enzymes, oxygen radicals, arachidonic metabolites, and cytokines, which in turn bring about inflammation and tissue damage in acute gout [5, 7, 8]. It has been known that MSU can upregulate the expression of proinflammatory cytokines and help to propagate a local or systemic inflammatory process. Cytokines, such as tumor necrosis factor-alpha (TNF-*α*), interleukin-1 beta (IL-1*β*), and interleukin-8 (IL-8), have been found in symptomatic joints of arthritic patients and have been implicated in acute gouty arthritic [3, 5–10]. The goals of gouty arthritis treatment are anti-inflammatory therapy to manage the significant pain, swelling, and disability associated with acute attacks [11, 12]. Nonsteroidal anti-inflammatory drugs (NSAIDs) or colchicine is a first-line systemic treatment according to the current gouty arthritis treatment guidelines recommendation; glucocorticoids also have long been used to treat acute gouty arthritis attacks in patients who are intolerant to NSAIDs or colchicines [3, 13, 14].

In China, besides synthetic chemical medicine, herbal therapy is another effective alternative to treat gout and related gouty arthritis [15, 16]. RuPeng15 (PRP15) is a herbal multicompound remedy, comprised of 15 specific herbs that originate from traditional Tibetan medicine and possess antigout, anti-inflammatory, and antihyperuricemic properties based on traditional concepts, which has been successfully practiced in Tibet for many centuries [17–19]. Clinical observation by* Cairang-LaQing *reported that PRP15 could significantly improve the symptoms and signs of 100 patients with rheumatoid arthritis, and our previous studies in animals showed the evidence that this remedy has anti-inflammatory and antinociceptive effects [20–22]. However, PRP15 has not been validated for the treatment of acute gouty in clinical studies or animal models of arthritis. As a first step, we want to investigate the efficacy of PRP15 against MSU crystal-induced gout in rat, an experimental model for gouty arthritis that is similar to those observed in clinical gout. Furthermore, we focus on the effect of PRP15 on the induction of inflammatory cytokines TNF-*α*, IL-1*β*, IL-8, and nuclear transcription factor-*κ*B p65 (NF-*κ*B p65) to investigate its anti-inflammatory mechanism.

## 2. Materials and Methods

### 2.1. Plant Material and Preparation

The RuPeng15 Powder (RPP15), a herbal mixture comprised of 15 ingredients: Indian frankincense (*Boswellia serrata*) 150 g,* Tinospora* spp. 150 g,* Cassia tora *120 g,* gypsum slag *75 g,* HuangKuizi *120 g,* Acorus calamus (Tibetan subspecies) *120 g,* Justicia adhatoda *110 g,* Acacia catechu *75 g,* Terminalia chebula *150 g,* Styrax benzoin *60 g,* MaoHezi *150 g,* Aconitum pendulum Busch *75 g,* Saussurea lappa *150 g, musk (*Moschus* spp.) 1.5 g, and* Phyllanthus emblica* 150 g were gotten from the Qinghai Provincial Tibetan Medical Hospital, the authority in the area on Tibetan medicine. According to Tibetan doctors, the recommended dosage of RPP15 for adults was 2.4 g (total raw materials/day). In rats equivalent doses were about 7 times the human dose, respectively. Based on clinical observation of the safety of the drug, we chose 10, 20, and 30 times the human dose as lower, middle, and high dosage, respectively. Three doses of RPP15 were used at 0.4 g/kg, 0.8 g/kg, and 1.2 g/kg suspended in distilled water and administrated by oral gavage for 8 days in the study. Indomethacin 3 mg/kg (Yunpeng, ShanXi, China) as a positive control was similarly suspended in distilled water.

### 2.2. Animals

The male SD rats (220–240 g) used for the experiment were obtained from Gansu Traditional Chinese Medicine College, China (certificate of quality: SCXK-2004-0006). All animals were maintained on a 12 h day-night cycle and the temperature and humidity were kept at 22–24°C and 50%, respectively, with freely available food and water. All possible efforts were made to minimize animal suffering and reduce the number of animals used according to the recommendation of the local and national ethic committees, and no animal died during the experiment.

### 2.3. MSU Preparation and the Gout Animal Model

MSU crystals (from sigma) were sterilized by heating at 180°C for 2 h before experiments. Rats were anesthetized with intraperitoneal injection at a dose of urethane (1.0 g/kg), and MSU crystals (20 mg/mL) were injected into the synovial space of both sides of the knee joint in a volume of 50 uL sterile phosphate buffered saline (PBS) before the injection the suspensions was vortexed vigorously [6].

### 2.4. Experimental Design

Rats were allocated randomly to 6 groups, each comprising of ten rats. Group I served as a control group treated by vehicle and received an ia injection of sterilized PBS in the knee. In group II (Model group), gouty arthritis was induced by MSU crystal and treated by vehicle. Group III monosodium crystal-induced rats were treated with indomethacin (3 mg/kg) as positive control group. Group IV–VI is comprised of MSU crystal-induced rats which were treated with RPP15 (0.4, 0.8, and 1.2 g/kg). The freshly prepared RPP15 (0.4, 0.8, 1.2 g/kg) and indomethacin were oral administered once a day for 8 days. Before administration, the suspensions were vortexed vigorously. On the seventh day, arthritis was conducted at one hour after test compounds administration. The development of arthritis was assessed by measuring the size of the joint with vernier caliper at 0 h (baseline, before the ia injection), 2 h, 4 h, 6 h, 8 h, 12 h, and 24 h after the MSU crystal injection. At 24 hours after the induction of gout, the rats were sacrificed by overdose of anesthesia; synovial lavage fluid from each knee was collected by injecting 1 mL PBS into the joint cavity. Lavage fluids were then centrifuged at 400 g for 10 minutes and supernatants were stored at −80°C before biochemical determinations, and synovial tissue was gently separated for histopathology examination.

### 2.5. Histologic Evaluation and Immunohistochemistry

Synovial tissue was preserved in 10% phosphate buffered formaldehyde, dehydrated in ethanol series, and embedded in paraffin. Sections were stained with hematoxylin and eosin. Histopathological examination was used to determine the number of infiltrating cells and to characterize the neutrophil and leucocyte populations present. The expression of NF-*κ*B p65 was investigated by immunohistochemistry analysis of 5 *μ*m paraffin sections. Briefly, sections were deparaffinized with xylene, dehydrated using a graded ethanol series before the heated, mediated antigen retrieval with 10 mM citrate buffer, pH 6, and then washed with phosphate buffered saline and blocked endogenous peroxidase activity by 0.3% H_2_O_2_ for 30 min at room temperature. After another three rinses with PBS, the tissues were blocked with 5% BSA for 30 min at 37°C and incubated with rabbit primary anti-NF-*κ*B p65 antibody (dilution, 1 : 500, Abcam) for 16 hours at 4°C. After three rinses with PBS, the sections were incubated with goat anti-rabbit IgG for 2 hours and incubated with avidin-biotin peroxidase in combination with an ABC kit (Wuhan Boster Bioengineering Institute, China). Finally, after three times of washing, the sections reacted with 3,3′-diaminobenzidine tetrahydrochloride (DAB). All slides for immunochemical staining were analyzed using an upright microscope (Olympus BX53, Japan) in combination with a digital image output system (Mshot, Guangzhou Mingmei Technology Co. Ltd., China). An Information Management System (IMS) cell image analysis system was used for quantitative analysis. Five fields of view were randomly selected from each slice, and an index of positive staining was determined from the integrated optical density and the area of positive staining. The positive index was calculated as MOD, mean optical density.

### 2.6. Measurement of Inflammatory Factors in the Knee Joint Lavage Fluid

The protein levels of TNF-*α*, IL-1*β*, and IL-8 in the knee joint lavage fluid, which could be secreted during the process of acute gouty arthritis induced by MSU, were determined using commercially available enzyme-linked immunosorbent assay (ELISA) kits in accordance with the manufacturer's instructions: TNF-*α*, IL-1*β* ELISA kits purchased from Sigma-Aldrich, USA; IL-8 was gotten from Nanjing Jiancheng Bioengineering Institute, China.

### 2.7. Statistical Analyses

All data were expressed as mean ± SD. For statistical analysis, all of the data were analyzed using a one-way analysis of variance, followed by post hoc Dunnett's test. Data of knee swelling were analyzed using a repeated ANOVA. *P* < 0.05 was considered statistically significant.

## 3. Results

### 3.1. Effects of RPP15 on Knee Swelling in MSU Crystal-Gouty Arthritis Rats

**Figure 1:**
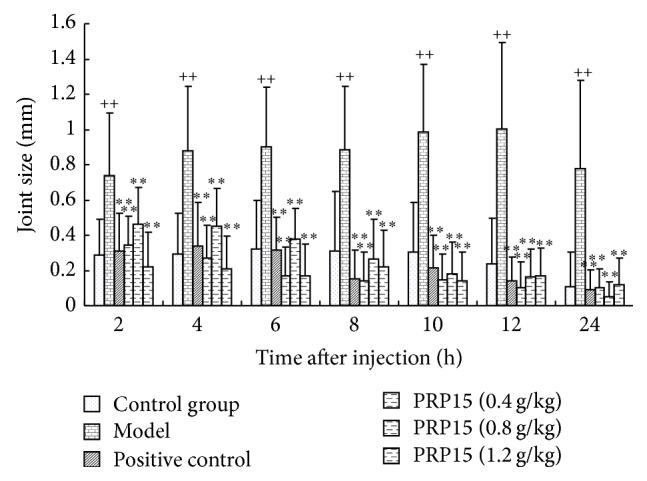
Effects of RPP15 on knee swelling in MSU crystal-induced gouty arthritis rats. Rats were pretreated with either vehicle, RPP15 (0.4, 0.8, and 1.2 g/kg, p.o.), or positive drug indomethacin (3 mg/kg). Knee joint swellings were evaluated at different time points. Data are means ± SD (*n* = 9). ^++^ *P* < 0.01 versus vehicle-control group (control); ^*∗∗*^ *P* < 0.01 versus vehicle with MSU crystal (model).

As shown in Figure 1, a repeated ANOVA with MSU crystal as independent factor and hour as repeated factor revealed that between the different times the swelling degree of rat knee joint has been statistically different (*P* < 0.05), and in the model group gouty arthritis induced by MSU crystal joint swelling was significantly higher than the control group (*P* < 0.01), and MSU crystal and hour have no interaction (*P* > 0.05). Then, we took treatment as independent factor and hour as repeated factor in rats with gouty arthritis induced by MSU; the results demonstrated that, with the treatment of 0.4 g/kg RPP15, 0.8 g/kg RPP15, 1.2 g/kg RPP15, and 3 mg/kg indomethacin, the joint swelling decreased significantly compared to gouty arthritis rats treated with vehicle (*P* < 0.01); further, the statistics analyzed by one-way ANOVA showed such differences that existed in all time points 2 h, 4 h, 8 h, 12 h, and 24 h after the MSU crystal injection (*P* < 0.01).

### 3.2. A Histomorphometric Analysis of the Effect of RPP15 on MSU-Induced Infiltration

**Figure 2:**
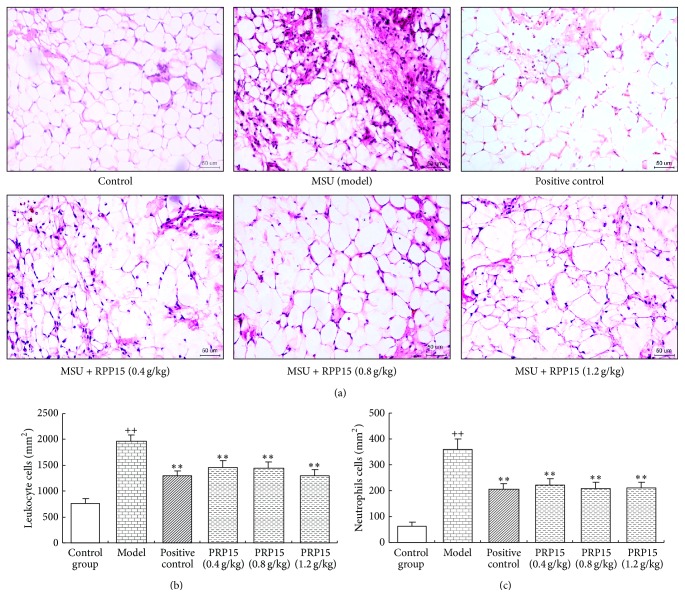
Histomorphometric analysis of the effect of RPP15 on MSU-induced leukocyte and neutrophil infiltration. (a) Representative histological changes using HE stain of synovial tissue from rats knee joint 24 h after the injection of MSU crystals were shown (magnification, ×200). (b) Leukocyte and (c) neutrophil infiltration number were counted from HE stain of synovial tissue slides (cells per square millimeter). Data are means ± SD (*n* = 6). ^++^ *P* < 0.01 versus vehicle-control group (control); ^*∗∗*^ *P* < 0.01 versus vehicle with MSU crystal (model).

Synovial specimens were obtained from the inflamed knee joints in rats at 24 h after injection with MSU crystals. These specimens were then stained with hematoxylin and eosin. MSU crystal significantly increased leukocyte infiltration (primarily neutrophils) of the superficial synovium compared with that in control group. Cotreatment with indomethacin or RPP15 (0.4, 0.8, and 1.2 g/kg) inhibited leukocyte infiltration as shown in Figure 2(a). As shown in Figures 2(b) and 2(c), compared with control group, the number of leucocytes and neutrophils was significantly elevated in MSU model groups, and in the the groups treated with indomethacin (3 mg/kg) and RPP15 (0.4, 0.8, and 1.2 g/kg) the number of leucocytes and neutrophils was significantly reduced compared with model group (*P* < 0.01).

### 3.3. Effects of RPP15 on NF-*κ*B p65 Protein Expression in Synovium Sections

**Figure 3:**
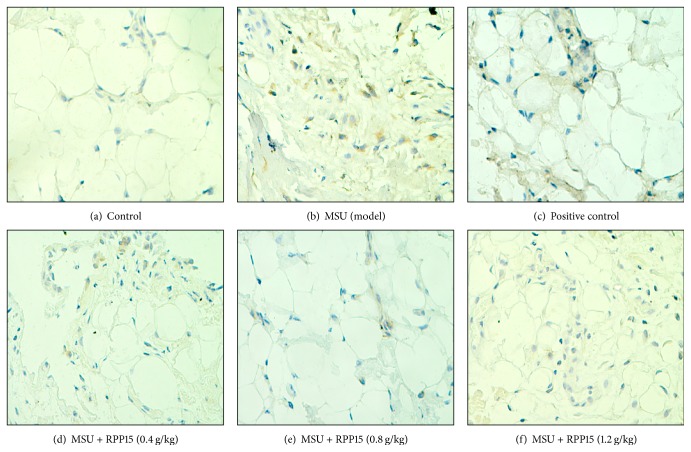
The Illustrated levels of knee synovium NF-*κ*B p65 immunoreactivity in control (Figure 3(a), ×400), MSU (Figure 3(b), ×400), MSU + indomethacin (Figure 3(c), ×400), and MSU + RPP15 (0.4, 0.8, 1.2 g/kg,) rats (Figures 3(d), 3(e), and 3(f), ×400).

The effect of RPP15 treatment on expression of NF-*κ*B p65 in the synoviumsections of MSU-induced rats was assessed by immunohistochemistry. Representative synovium sections from each treatment group were shown in Figure 3 and quantitative analysis is presented in Table 1. Immunohistochemical results showed that in MSU crystal-induced gouty arthritis rats pretreated with vehicle (model) the positive expression of NF-*κ*B p65 in the synovium sections was significantly increased compared with control group (*P* < 0.01) (Figure 3(b), Table 1) and NF-*κ*B p65 expression was observed in the cytoplasm and the nucleus. However, in the groups treated with RPP15 (0.4, 0.8, and 1.2 g/kg) or indomethacin (3 mg/kg), the expression of NF-*κ*B p65 was significantly decreased when compared with that in the model group (*P* < 0.01) (Figures 3(c)–3(f), Table 1).

### 3.4. Effect of RPP15 on MSU Crystal-Induced Inflammatory Mediator Production in the Knee Joint Lavage Fluid

**Figure 4:**
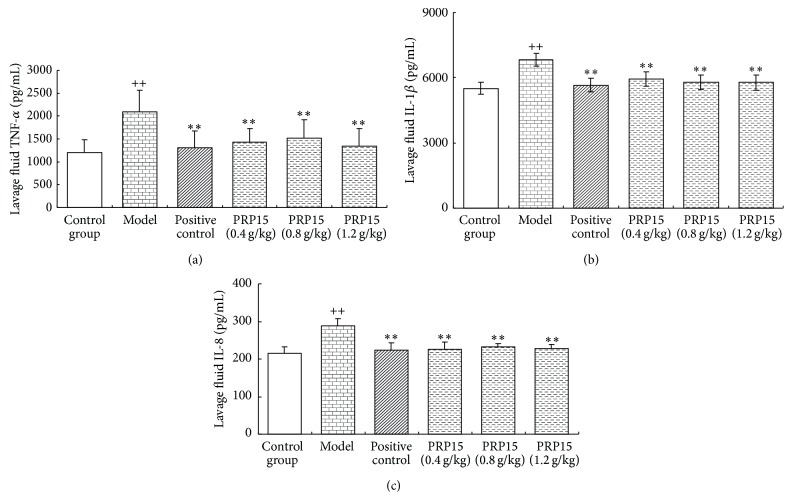
Effect of RPP15 on MSU crystal-induced inflammatory mediator production in the knee joint lavage fluid. To examine the therapeutic potential of RPP15 on gout, cytokines- TNF-*α*, IL-1*β*, and IL-8 were measured 24 h after MSU crystal. Data are means ± SD (*n* = 6). ^++^ *P* < 0.01 versus vehicle-control group (control); ^*∗∗*^ *P* < 0.01 versus vehicle with MSU crystal (model).

To examine the effect of RPP15 on inflammatory mediator production induced by MSU, TNF-*α*, IL-1*β*, and IL-8 levels were determined in the knee joint lavage fluid. MSU injection significantly increased joint lavage fluid, TNF-*α* (Figure 4(a)), IL-1*β* (Figure 4(b)), and IL-8 (Figure 4(c)) levels, compared with that in control group (*P* < 0.01). RPP15 (0.4, 0.8, and 1.2 g/kg) significantly decreased all tested proinflammatory cytokine production in joint lavage fluid compared with that in MSU alone group (*P* < 0.01) (Figures 4(a)–4(c)).

## 4. Discussion

Gout is an autoinflammatory disease associated with increased blood levels of urate due to deposition of monosodium urate crystals in and around joints. Over recent decades, the prevalence of hyperuricemia is steadily increasing and gout is becoming one of the most common causes of inflammatory arthritis [23]. In China, the adjusted prevalence of hyperuricemia among Chinese adults in 2009-2010 is 8.4% the, but it also varies across different populations and different areas [24]. There is a study that shows that the incidence rate of hyperuricemia in Tibetan population in Qinghai Tibet Plateau is much higher than the Han nationality in plain area. This may be associated with the Tibetan people living in the cold climates, loving to eat beef and mutton, and enjoying drinking the wine alcohol [25]. So, in the fighting against the disease, they form unique therapy theory and medicine called Tibetan medicine, which, with a history going back approximately to 2,500 years, is known as one of the world's oldest known traditional medicines. It is beneficial for chronic diseases such as digestive problems, arthritis, gout, asthma, and problems related to the liver, kidneys, and heart [26]. As a traditional medicine, the future development of Tibetan medicine is linked to being recognized as a popular and viable healthcare option providing an alternative clinical reality.

Assessing and treating patients of gout Tibetan medicine has a long history [27]. RuPeng15 Powder (RPP15) is a classic Tibetan medicine in the treatment of gout that recorded by “Tibetan Notes,” a text compiled by Wendeng Jiacuo Gong Zhu in 1813 having antigout, anti-inflammatory effects in the theory of Tibetan medicine [17–19]. Although RPP15 has been seen to be an effective and safe prescription in the treatment of gout for centuries, Tibetan populations have used it based on classical records and clinical experience. Its functional roles in the treatment of gout have not been completely revealed. In our present study, we assessed the antigouty arthritis effect of the RPP15 in rats with MSU crystal-induced gouty arthritis. The study showed that RPP15 treatment (0.4, 0.8, and 1.2 g/kg) reversed MSU crystal-induced elevation in knee swelling compared with the model group. In addition, according to the histopathological change of knee synovium analysis, it was suggested that RuPeng15 was able to attenuate the MSU-induced infiltration of neutrophils and leukocytes. These results demonstrated that RuPeng15 possesses certain anti-inflammatory effects.

The potential for MSU crystal-induced rat joint inflammation as a valid experimental model of gouty arthritis was determined by testing classic antigout drugs [6, 28]. This experimental model caused a remarkable accumulation of neutrophils that is similar to the situation in humans. Neutrophil influx peaked at 16 hours and being consistent with the elevation of IL-1*β*, IL-8, and TNF-*α* release, which could be detected subsequently by 24 h after the induction of arthritis. Interleukin- (IL-) 1*β*, IL-8, and tumor necrosis factor- (TNF-) *α* are important cytokines in inflammation and are considered to be the most important mediators involved in the pathogenesis of gout that recruit neutrophils to the site and start the inflammatory cascade [3, 5–10, 23]. A number of these cytokines are not randomly present; their expression is controlled by a network or hierarchy which activates the expression of nuclear transcription factor- (NF-) *κ*B. NF-*κ*B is clearly one of the most important regulators of proinflammatory gene expression that can upregulate the expression of lots of cytokines such as TNF-*α*, IL-1*β*, and IL-8 [29, 30]. The activated NF-*κ*B is reported to form a heterodimer, which usually consists of two proteins, p65 and p50 subunits. The p65 subunit has been demonstrated to exert critical activity on the transcription of many inflammatory genes, including adhesion molecules, cytokines, and chemokines [31, 32]. Therefore, the downregulation of NF-*κ*B signaling pathway may be an effective and rational approach for the treatment of gout.

In the present study we demonstrated that in MSU crystal–induced rat model of gouty arthritis RPP15 (0.4, 0.8, 1.2 g/kg) significantly downregulated the inflammatory factors (TNF-*α*, IL-1*β*, and IL-8) levels in the knee synovial fluid, and immunohistochemistry analysis indicated RPP15 (0.4, 0.8, 1.2 g/kg) suppressed the expression of active NF-*κ*B p65 in the synovium.

## 5. Conclusion

In conclusion, antigout effects of RPP15 were observed in rats with MSU-induced gouty arthritis in which RPP15 inhibited joint swelling and suppressed inflammatory cell infiltration. This beneficial antigouty arthritis effect might be mediated, at least in part, by inhibiting TNF-*α*, IL-1*β*, IL-8, and NF-*κ*B p65 protein expression in synovial fluid and synovial tissue, and the suppression of NF-*κ*B might be responsible for the decrease of the levels of IL-6, IL-8, and TNF-*α* in synovial tissue.

## Figures and Tables

**Table 1:** MOD values of NF-*κ*B p65 in the synovium (mean ± SD).

Group	NF-*κ*B p65
Vehicle + control group	0.026 ± 0.006
Vehicle + MSU crystal	0.097 ± 0.022^++^
MSU + indomethacin (3 mg/kg)	0.047 ± 0.013^*∗∗*^
MSU + PRP15 (0.4 g/kg)	0.046 ± 0.003^*∗∗*^
MSU + PRP15 (0.8 g/kg)	0.038 ± 0.013^*∗∗*^
MSU + PRP15 (1.2 g/kg)	0.036 ± 0.016^*∗∗*^

For quantitation, 30 visual fields were analyzed from each group, five from each of the six sections from separate rats. From these, the MOD values were calculated. MOD: mean optical density. Data are means ± SD (*n* = 6). ^++^ *P* < 0.01 versus vehicle-control group (control). ^*∗∗*^ *P* < 0.01 versus vehicle with MSU crystal (model).
